# Synthesis of Pyridine Heterocyclic Low-Melting-Point Phthalonitrile Monomer and the Effects of Different Curing Agents on Resin Properties

**DOI:** 10.3390/polym14214700

**Published:** 2022-11-03

**Authors:** Xinyang Zhang, Xinyang Wang, Jian Li, Shuo Zhang, Qingxin Zhang, Xiaoyan Yu

**Affiliations:** 1 Hebei Key Laboratory of Functional Polymers, School of Chemical Engineering and Technology, Hebei University of Technology, Tianjin 300130, China; 2 School of Chemical Engineering and Technology, Tianjin University, Tianjin 300130, China

**Keywords:** phthalonitrile resin, processing window, low melting point, long-term stability

## Abstract

A phthalonitrile monomer (DPTP) containing pyridine with sulfide bonds was prepared and cured into polymers using different curing agents under the same temperature-programmed process. We characterized and comprehensively evaluated the effects of different curing agents on the thermal and thermomechanical properties of phthalonitrile resin, showing that the DPTP monomer cured with naphthalene-containing curing agent exhibited the best performance among the three polymers. Differential scanning calorimetric (DSC) investigation manifested that the melting point of the DPTP monomer was 61 °C, with a processing window of about 170 °C, suggesting the presence of a wide processing range. Thermogravimetric analysis (TGA) demonstrated the outstanding heat resistance, *T*_5%_, of 460 °C in nitrogen, at the same time demonstrating superior long-term stability compared with other commonly used polymer materials, which proves the long-term usage under high temperatures of 300 °C. Dynamic mechanical analysis (DMA) revealed that the storage modulus at 50 °C was 3315 MPa, and the glass transition temperature (*T*_g_) of the polymer was more than 350 °C. Therefore, DPTP resins have favorable thermal stability as well as prominent thermomechanical properties.

## 1. Introduction

Polymer materials are widely used in transportation, communication, construction, and other social fields of the modern era, and play a vital role in military and civil industries and across all walks of life. High-performance polymers are a class of thermally stable polymeric materials with high melting points, and which can be used for long periods of time at temperatures above 300 °C. Common high-performance polymers include polyimide, polybenzoxazine, polyether ether ketone (PEEK), phthalonitrile (PN), etc. [1,2,3,4,5,6,7,8]. The requirements for high temperature resistance, corrosion resistance, and high flame retardancy of materials are increasing. As a high-temperature thermosetting polymer originally developed by the Naval Research Laboratory, PN resin has widely attracted attention due to its superior performance, such as favorable high temperature resistance and preeminent thermal and thermo-mechanical properties [9,10,11,12], and it has great development space and important application value in the aerospace industry and electronic packaging (microelectronics), shipbuilding, and other fields [13,14,15,16,17,18]. Nevertheless, the PN monomer has a high melting point (*T*_m_) and curing temperature, with a strict processing process and narrow processing window (temperature difference between *T*_m_ and curing temperature), requiring curing for a long time at higher temperature. The applications are greatly limited in industry, and improving the performance of the PN monomer is still a huge challenge.

Efforts to solve the problems of processability and functionalities have centered on the design and development of monomer molecular structures. The performance of PN resin can be adjusted through molecular design. In particular, introducing heterocyclic rings and high-volume rigid naphthalene ring structures can improve the polymer properties.

First, the protonation of polar nitrogen atoms on the nitrogen-containing heterocyclic ring (such as the pyridine ring, pyrazine ring, and pyrimidine ring) can increase the solubility of polar solvents; in addition, nitrogen-containing heterocyclic rings have greater polarity than benzene rings, which can reduce the polarizability of the polymer after the introduction of the main chain, which lowers the dielectric constant. Meanwhile, nitrogen heterocycles have symmetrical structures, better rigidity, and thermal stability; thus, their introduction through molecular design can improve the thermal stability and thermomechanical properties of the materials [19]. Furthermore, the nucleophilic reactance reaction occurs easily because the high polarity of the nitrogen atom decreases the electron density of the carbon atom. Secondly, when the naphthalene ring is introduced into the main chain, due to the rigid plane structure of the naphthalene ring, the rigidity of the polymerization can be increased, and the glass transition temperature (*T*_g_) and service temperature of the polymer can be improved. The polymer can be further modified using the higher reactivity and more chemical reaction sites of the naphthalene ring. 

Based on molecular design, the *T*_m_ of PN monomer can be reduced by introducing flexible aryl ether bonds, and the processing window of the monomer can be increased. However, the introduction of flexible chain segments will lead to the reduction in crosslinking density. Thereby, rigid aromatic heterocyclic groups and naphthalene can be introduced into the framework of monomer to improve the thermal stability and thermal-mechanical properties of PN resin. At present, to acquire PN resin with superior performance, diverse nitrogen heterocyclic-containing and naphthalene-containing PN monomer structures have been composed [20,21,22,23,24,25]. Xi et al. [19] first introduced pyridine rings into phthalonitrile resins. The thermal stability was excellent, with *T*_5%_ greater than 530 °C. The resin also had excellent thermomechanical properties, with *T*_g_ greater than 400 °C. However, the monomer exhibited a high melting point of 191 °C and a narrow processing window. Yang et al. [21] successfully prepared a phthalonitrile resin containing a pyrimidine ring, which exhibited good properties due to the introduction of the pyrimidine ring, The energy storage modulus reached approximately 1700 MPa at 400 °C. Additionally, the processing performance of the monomer was still not good enough, with a processing window of about 117 °C. Two PN resins containing vinyl pyridine rings were prepared by Wang et al. [22]. The *T*_g_ of both PN resins reached above 400 °C. Liu et al. [23] prepared a monomer containing a pyrazine ring by a one-pot, two-step method with a low reaction activation energy of 32.57 kJ mol^−1^. The processing window of the monomer was about 94 °C, exhibiting limitations to the processing properties. Recent research has shown that inter- and intramolecular reactions are in competition and are key in the final properties. Changing the reaction conditions can complicate the competition between inter- and intra-molecular reactions and their interactions with diffusion limitations, as shown by experiments simulating changes in hydrophilicity by Keer et al. [26]. This can affect the localization pattern of the functional groups, and thus, the final properties. The study by Jin et al. [27] showed a shift in branching density under NCO conversion, their model demonstrating which intermolecular reactions can produce a three-dimensional network and unreacted functional groups which can be converted into active functional groups by introducing intramolecular reactions, resulting in a more realistic polymer network. The choice of curing agent, the length of curing time, and the level of curing temperature will affect the properties of PN resin. A comprehensive understanding of the curing mechanism of PN resin is important for optimizing the processing conditions of the resin, selecting the appropriate curing agent, synthesizing new PN monomers, and obtaining PN resin with outstanding properties. PN resins are normally formed by bulk addition polymerization of metal salts and Lewis acids, aromatic organic compounds embodying active hydrogen atoms such as amino and hydroxyl groups as curing agents with monomers under high-temperature conditions [28,29,30,31,32]. The addition cure mechanism evolved neither solvents nor volatiles during polymerization, producing highly cross-linked, void-free resin under the action of specific curing agent; the cyano group in PN monomer formed a stable cross-linked network structure. It has been shown that the curing agent containing the cyano group has a better curing effect, and the resin it produces has higher thermal stability and thermomechanical properties compared with other curing agents. With the increase in cyano density, the polymer is cross-linked more closely and the polymerized degree is greater [33,34].

In this study, based on previous research, the pyridine ring was introduced to give the resin excellent thermal stability and better thermomechanical properties. The introduction of the flexible chain segment of the thioether bond into the structure served to lower the melting point of the monomer while improving its processing properties. Additionally, using different curing agents, the naphthalene ring was introduced into the curing agent to investigate its effect on the monomer properties. 4,4-dihydroxydiphenyl sulfide, 2,6-dichloropyridine, and 4-nitrophthalonitrile were used as raw materials to prepare the 4-(4-((4-((6-(4-((4-(3,4-diisocyanophenoxy)phenyl)thio)phenoxy)pyridin-2-yl)oxy)phenyl)thio)phenoxy) phthalonitrile monomer (DPTP) with pyridine structure. The composition, structure, and characteristics of the DPTP monomer and polymers which were cured by 4,4′-diaminodiphenyl ether (ODA), 4-(aminophenoxy) phthalonitrile (APPH), and 4-((8-Aminonaphthalen-2-yl) oxy) phthalonitrile (ANP) as curing agents to cure DPTP at programmed temperature were determined. The pyridine structure and thioether bond flexible chain segment were introduced in the PN monomer. Due to the long molecular chain and satisfactory flexibility, the *T*_m_ was reduced and the processing window enlarged, thus improving the processing performance. To enhance the thermal stability and thermomechanical properties of PN resin, various curing agents were used and naphthalene rings were introduced into the curing agent to improve the thermal stability and mechanical and water absorption properties, including rigid naphthalene rings the curing agent to explore their impact on the properties of PN resin. 

## 2. Experimental Section

### 2.1. Materials

4,4′-dihydroxydiphenyl sulfide (AR, 99.5%) was supplied by Tianjin Kaimate Chemical Co., Ltd., Tianjin, China. 2,6-dichloropyridine (AR, 98.0%) was purchased from Shanghai Dibai Chemical Technology Co., Ltd., Shanghai, China. 4-nitrophthalonitrile (AR, 98.0%) was provided by Zhengzhou Alpha Chemical Co., Ltd., Jiaozuo, China. N, N-dimethylformamide (DMF, 99.5%) and toluene (AR, 99.0%) was acquired from Tianjin Fuchen Chemical Co., Ltd., Tianjin, China and Tianjin Hengshan Chemical Technology Co., Tianjin, China, respectively. Anhydrous potassium carbonate (K_2_CO_3_, 99.0%) was produced by Shanghai Aladdin Reagent Co., Ltd., Shanghai, China. 8-Amino-2-naphthol (AR, 95.0%) Shanghai DB Chemical Technology Co., Ltd., Shanghai, China. 4,4′-diaminodiphenyl ether (ODA) (AR, 98.0%) was obtained from Shanghai Bide Pharmaceutical Co., Ltd., Shanghai, China. 4-(aminophenoxy) phthalonitrile (APPH) was obtained according to the synthetic method synthesized and reported previously from 4-nitrophthalonitrile and 3-aminophenol in the lab [35]. The percentages of specifications are all mass fractions. All the chemicals were used without further purification.

### 2.2. Synthesis of ANP PN Monomer

A naphthyl-based self-promoted PN monomer, 4-((8-aminonaphthalen-2-yl) oxy) phthalonitrile (ANP), was successfully prepared via the nucleophilic substitution reaction through a one-pot reaction. First, 8-amino-2-naphthol (7.96 g, 50.00 mmol), 4-nitrophthalonitrile (8.66 g, 50.00 mmol), and anhydrous K_2_CO_3_ (6.91 g, 50.00 mmol) as acid binding agents were added to a 250 mL three-necked flask with DMF (60 mL) as the solvent; then, a condensing tube was connected to the flask. The flask was placed in an oil bath, and the reaction mixture was stirred and refluxed at 80 °C for 8 h until the reaction was complete. After the heating was stopped and the reaction was completed, the mixed solution was subjected to decompression distillation at 80 °C to remove DMF in the solution until the reaction solution became sticky. The reaction solution was poured into 1600 mL of deionized water and soaked overnight. The dark gray precipitate collected by filtration was washed repeatedly with excess deionized water by suction filtration until the filtrate was neutral. The obtained precipitate was dried in a vacuum oven at 55 °C for 48 h, and the yield was 90.3%. 

### 2.3. Preparation of DPTP PN Monomer and Polymers 

The monomer was synthesized by nucleophilic substitution, and the reaction was divided into two main stages. The first stage is the dehydration stage, also known as the salt formation reaction, in which weakly basic potassium carbonate is first ionized in the solvent to form potassium ions (K^+^), and then undergoes a salt formation reaction with the hydroxyl group to produce the potassium salt of phenol, along with water and carbon dioxide by-products. The phenol potassium salt then undergoes a nucleophilic substitution reaction with halogen, also known as the desalting reaction, to produce a potassium salt intermediate, which then undergoes a nucleophilic reaction with 4-nitrophthalonitrile to produce the target monomer (DPTP).

First, the reaction took place in a 250 mL three-necked flask containing 4,4′-dihydroxydiphenyl sulfide (10.00 g, 45.80 mmol), 2, 6-dichloropyridine (3.39 g, 22.90 mmol), anhydrous potassium carbonate (K_2_CO_3_) (12.66 g, 91.60 mmol), DMF (80 mL), and toluene (10 mL), then connected to the Dean–Stark trap with toluene until it flowed back, and then attached to a condensate tube. The flask was placed in an oil bath at 160 °C, and the mixture was stirred and refluxed for 12 h until there were no water droplets flowing down. Once making sure that the compound reacted completely, the heating was stopped, and the mixed solution was vacuum-distilled at 60 °C to remove toluene from the solution. DMF has a higher boiling point than toluene; therefore, toluene vaporized first during vacuum distillation and was completely removed during the process. After that, 4-nitrophthalonitrile (7.93 g, 45.80 mmol) was added and stirred at 80 °C for 8 h. Then, DMF was removed at 85 °C by vacuum distillation until the reaction solution became viscous. When the product was cooled to ambient temperature, it was poured into 1600 mL of deionized water for overnight soaking and filtering to collect off-white precipitate. Subsequently, it was washed with distilled water repeatedly until the filtrate became neutral, the obtained precipitate was dried in a vacuum oven at 40 °C for 48 h, and the calculated yield was calculated to be 92.4%.

**Figure 1:**
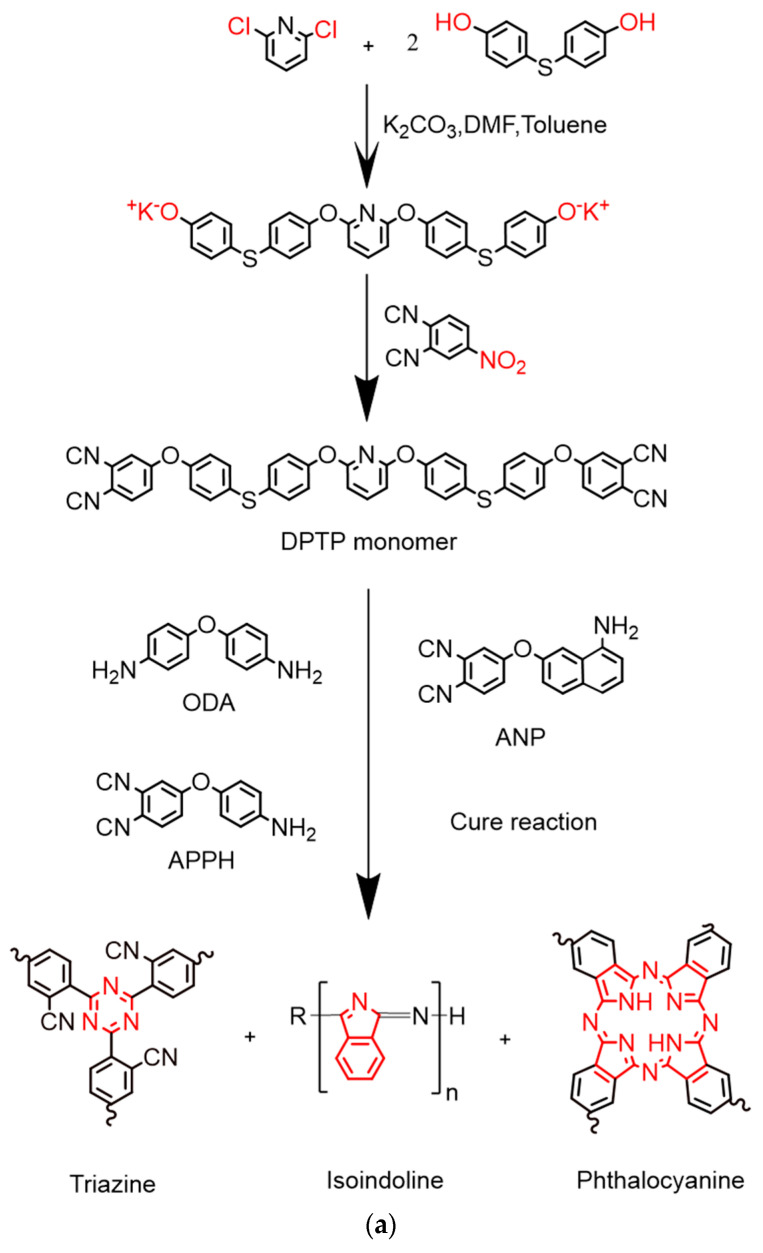

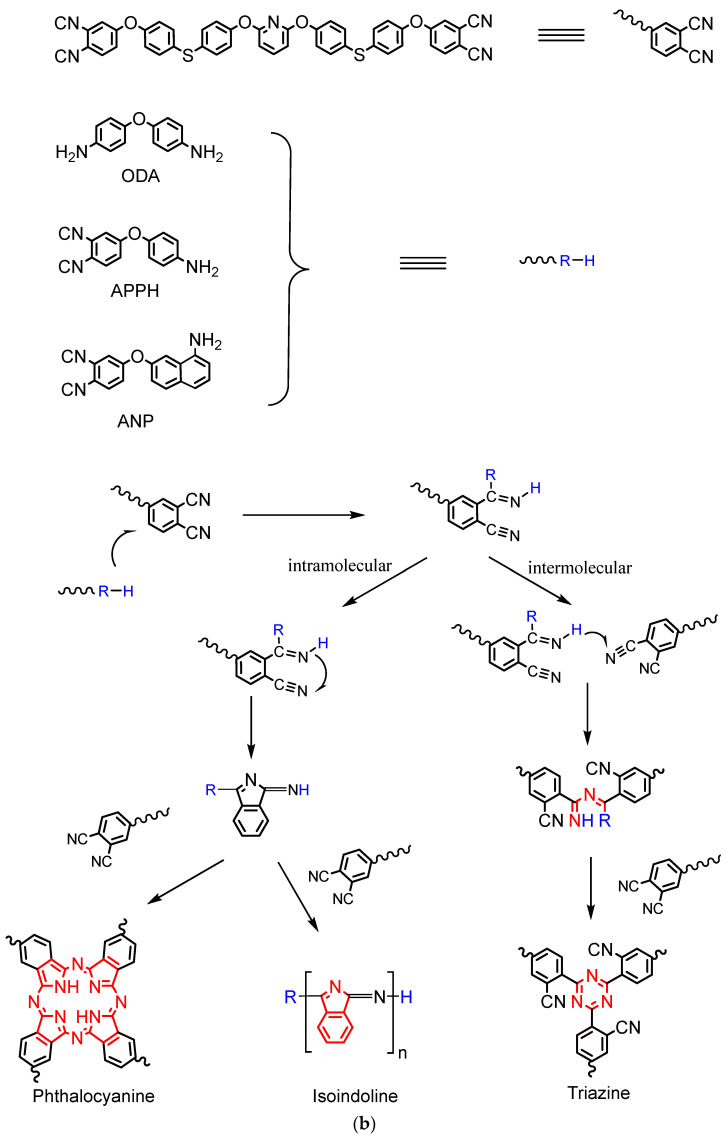
(**a**) Synthetic route of the DPTP monomer and polymer. (**b**) Curing mechanism of DPTP polymer.

DPTP monomers were cured with ODA5%, APPH10%, and ANP10% as curing agents, marked Polymer 1, Polymer 2, and Polymer 3, respectively. Firstly, ODA (0.10 g), APPH (0.20 g), and ANP (0.20 g) were added into 2.00 g DPTP monomer, and the powder was thoroughly ground in a mortar to evenly mix it. The powder mixture was placed in a specific mold and then vacuumed and degassed in a 200 °C vacuum oven for approximately 60 min. As the viscosity of the mixture increased and it was observed that bubbles did not clearly appear, the mixture was quickly fed into the Muffle furnace for curing. DPTP polymers were prepared in a Muffle furnace in an air atmosphere through the following heating procedures shown in Table 1. After cooling to room temperature with a heating rate of 2 °C min^−1^ in a Muffle furnace after fully drying, the remaining polymers were used for test characterization. The synthesis route and curing mechanism are shown in Figure 1.

### 2.4. Characterization Method

Fourier-transform infrared spectroscopy (FTIR) IRAffinity-1S Shimadzu Production Co., Kyoto, Japan was used to analyze the structure of monomers and cured resins. The polymers were ground into powder; then, an appropriate amount of DPTP monomer and polymers were mixed in dried potassium bromide particles (KBr pellets), and ground thoroughly in a mortar before pressing. The method was performed using an FTIR spectrometer between 4000 and 400 cm^−1^ under an air atmosphere.

^1^H and ^13^C nuclear magnetic resonance (NMR) spectra were used to characterize the chemical structure of monomers. Moderate amounts of the samples were dissolved in deuterated dimethyl sulfoxide (DMSO-*d*_6_) and tetramethylsilane (TMS) as solvent and internal standard, respectively, and conducted by a Bruker AVANCE-400 Co., Berlin, Germany, NMR spectrometer at a proton frequency of 400 MHz with indirect detection probe and a carbon frequency of 100 MHz with a broadband probe. 

The wide-angle X-ray diffraction (WAXD) spectrum of DPTP polymers was measured on a MiniFlex600 system purchased from Rigaku Co., Tokyo, Japan, focusing on Ni-filtered Cu K_α_ radiation (λ = 0.15406 nm) under the conditions of 40 kV and 15 mA carried out in reflection mode. The collected scanning angle 2θ was between 5° and 60°, with the step size of 0.02° and scanning speed being 6°/min.

Differential scanning calorimetric (DSC) measurements were obtained by TA Q20 Co., New Castle, PA, USA, from 50 to 350 °C at various ramping rates with a nitrogen flow rate of 50 mL min^−1^ as protective gas, taking appropriate amounts of sample (3–5 mg) under the conditions of the ramping rate of 5, 10, 15, and 20 °C min^−1^.

Dynamic mechanical analysis (DMA) was performed using a Q800 Co., New Castle, PA, USA (TA Instruments) dynamic mechanical analyzer scanning after polishing the DPTP resin with a metallographic sample polishing machine into 40 mm × 8 mm × 2 mm rectangular splines. The thermo-mechanical properties of DPTP polymers were analyzed under the temperature range of 50–350 °C with a ramping rate of 5 °C min^−1^ and 1 Hz test frequency in a single cantilever mode. 

Thermogravimetric analyses (TGA) were performed on a TA Q600 Instruments thermogravimetric analyzer obtained from Co., New Castle, PA, USA, with an appropriate amount of sample (5–10 mg) and the thermal stability was characterized from 30 to 1000 °C at a ramping rate of 10 °C min^−1^ under the conditions of 100 mL min^−1^ nitrogen or air flowing rate, respectively.

All samples were dried in a vacuum drying oven before testing [36]. 

## 3. Results and Discussion

### 3.1. Structural Characterization of Monomers and Polymers

#### 3.1.1. Synthesis and Structural Characterization of ANP PN Monomer

^1^H and ^13^C NMR techniques were used for typifying the monomer structure. 

^1^H NMR (400 MHz, dimethyl sulfoxide DMSO-*d*_6_, δ, ppm): 8.11 (d, *J* = 8.87 Hz, 1H), 7.90–7.87 (m, 3H), 7.42 (dd, *J*_1_ = 2.37 Hz, *J*_2_ = 8.89 Hz, 1H), 7.28–7.15 (m, 3H), 6.72 (d, *J* = 7.41 Hz, 1H), 5.75 (d, *J* = 7.41 Hz, 1H).

^13^C NMR (100 MHz, dimethyl sulfoxide DMSO-*d*_6_, δ, ppm): 161.13, 150.35, 145.06, 136.85, 132.70, 131.35, 127.26, 123.74, 123.09, 122.38, 120.00, 117.16, 116.53, 115.90, 115.82, 113.01, 108.69, 108.41.

**Figure 2:**
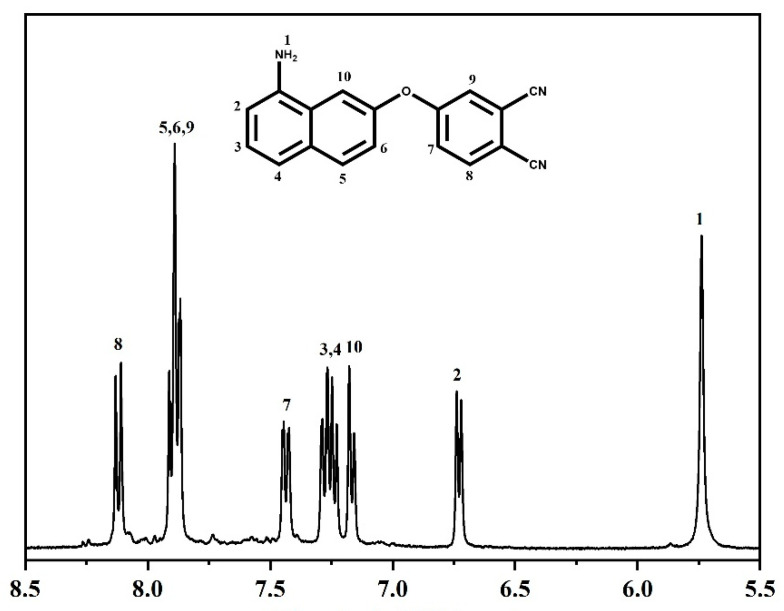
^1^H NMR spectrum of the ANP monomer.

In the ^1^H NMR spectrum, shown in Figure 2, the doublet peaks of proton 8 at 8.11 ppm were attributed to the orthodox coupling effect of proton 7. Due to the coupling effects of proton 8 and proton 9, a doublet peak at 7.42 ppm was observed for proton 7. The aromatic protons were detected at at the range of 6.72–8.11 ppm, and the resonance of proton number 2 was seen as a doublet at 6.72 ppm, with the signal of amino protons at 5.75 ppm.

**Figure 3:**
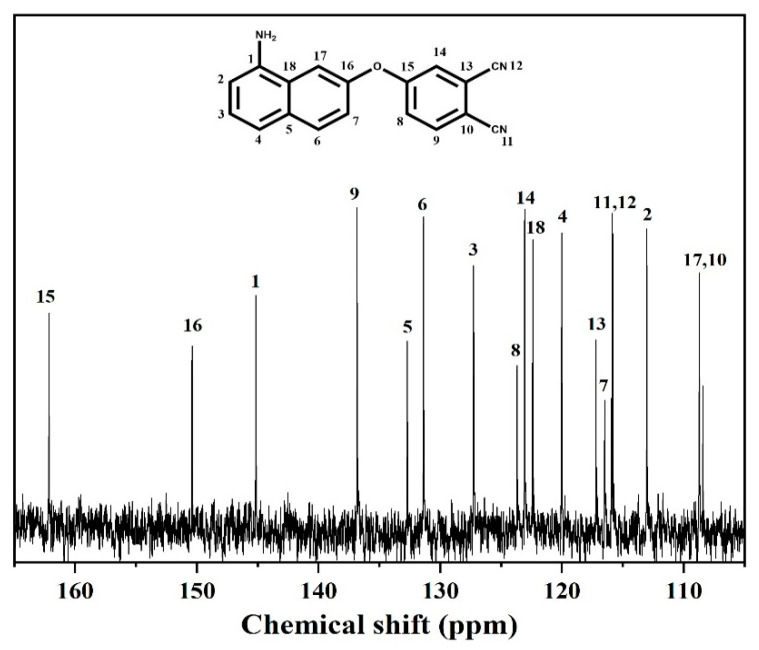
^13^C NMR spectrum of the ANP monomer.

In the ^13^C NMR spectrum (Figure 3), the signals at 108.41–161.13 ppm were assigned to the aromatic carbon atoms, except for those 115.90 and 115.82 ppm, which were due to the carbon atoms of cyano group. Hence, the obtained spectra agree well with the proposed molecular structure. 

#### 3.1.2. Structural Characterization of DPTP PN Monomer and Polymers

The synthesis of DPTP monomers can be divided into two stages. In the first stage, potassium carbonate reacts with phenolic hydroxyl as an acid-binding agent to generate phenolic potassium salt; then, phenolic potassium salt undergoes a nucleophilic substitution reaction with 2,6-dichloropyridine to introduce the pyridine nitrogen heterocyclic structure. In the second stage, phenolic potassium salt intermediate and 4-nitrophthalonitrile reacted to seal the end, and the PN monomer containing sulfide ether bonds was prepared. DMF was used as a solvent and toluene was the water-carrying agent. The monomer was characterized by ^1^H NMR, ^13^C NMR, and FTIR to determine its chemical structure.

**Figure 4:**
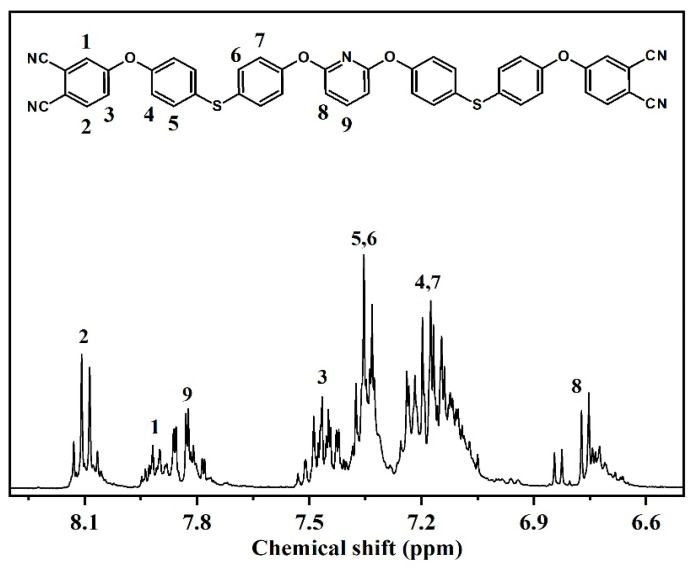
^1^H NMR spectrum of the DPTP monomer.

The ^1^H NMR spectra of DPTP monomer is shown in Figure 4. The multiple peaks at 8.13–8.07 ppm, 7.95–7.88 ppm, and 7.53–7.41 ppm are three hydrogen protons on the benzene ring of PN; multiple peaks at 7.39–7.23 ppm and 7.25–7.05 ppm correspond to hydrogen protons on the benzene ring; and multiple peaks at 7.85–7.77 ppm and 6.84–6.66 ppm represent hydrogen protons on the pyridine ring. 

**Figure 5:**
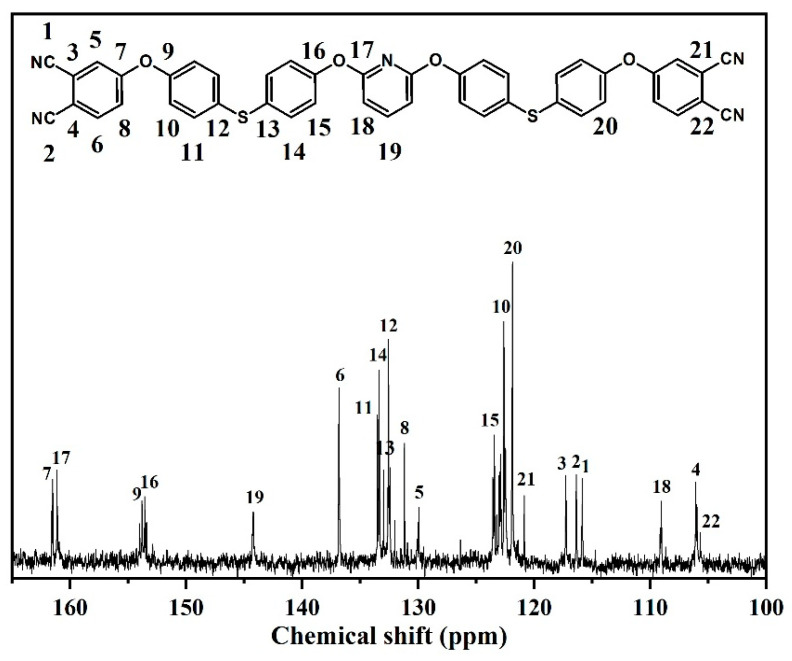
^13^C NMR spectrum of the DPTP monomer.

The ^13^C NMR spectrum of DPTP monomer is shown in Figure 5. The signals at 116.36 ppm and 115.85 ppm correspond to two cyano-based carbon protons on PN. The signals present at 161.14 ppm, 144.21 ppm, and 109.05 ppm represent carbon protons on the pyridine ring; the rest of the signals signify carbon protons on the benzene ring. Combined with the FTIR spectrum, it could be proven that DPTP is successfully synthesized, with the detailed data of chemical displacement as follows:

^1^H NMR (400 MHz, dimethyl sulfoxide DMSO-*d*_6_, δ, ppm): 8.13–8.07 (m, 2H; Ar-H), 7.95–7.88 (m, 2H; Ar-H), 7.85–7.77 (m, 1H; Ar-H), 7.53–7.41 (m, 2H; Ar-H), 7.39–7.23 (m, 8H; Ar-H), 7.25–7.05 (m, 8H; Ar-H), 6.84–6.66 (m, 2H; Ar-H).

^13^C NMR (100 MHz, DMSO-*d*_6_, δ, ppm): 161.52, 161.14, 153.77, 153.57, 144.21, 136.83, 133.50, 133.40, 133.00, 132.57, 131.19, 129.96, 123.42, 122.62, 121.84, 120.85, 117.26, 116.36, 115.85, 109.05, 106.05, 105.97.

**Figure 6:**
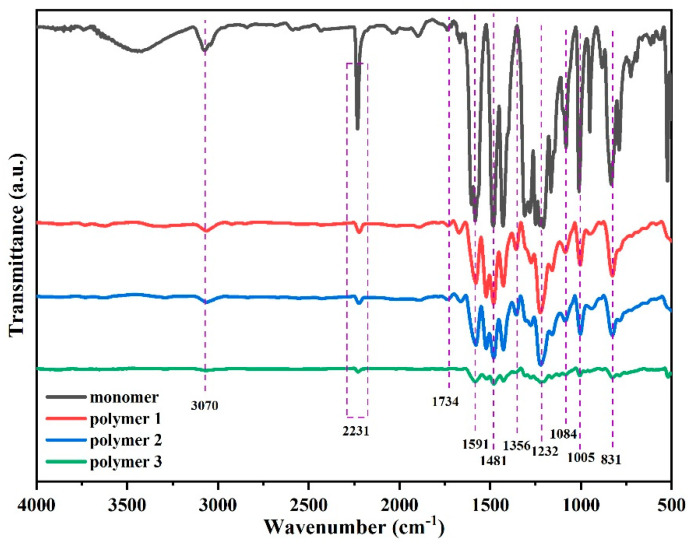
FT-IR spectra of DPTP monomer and polymers.

FTIR spectra of DPTP monomer and polymers are shown in Figure 6. The characteristic absorption peaks at 1084 cm^−1^ and 2231 cm^−1^ are attributed to the stretching vibration of the thioether bond and the cyano group (-CN), respectively, and the absorption signal at 1232 cm^−1^ corresponds to the stretching vibration of the aryl ether bond (AR–O–AR). The rest of the peaks are characteristic peaks on the benzene ring. The peak of the phenolic hydroxyl group (-OH) cannot be observed, indicating that nucleophilic substitution reaction occurred, and all -OH groups were replaced by 4-nitrophthalonitrile. The polymer mainly exists in triazine, phthalocyanine, and isoindoline [24,28]. For polymers, the absorption peak at 1005 cm^−1^ is attributed to the formation of the phthalocyanine ring structure, and two absorption peaks at 1734 cm^−1^ and 1481 cm^−1^ are typical isoindoline characteristic absorption peaks. It has been reported that based on the characteristic absorption peak of triazine at 1356 cm^−1^, it can be polymerized into a polytriazine structure. Compared with the IR spectra of the monomer, the characteristic absorption peak of the cyano group (-CN) at 2231 cm^−1^ has only a small residual, indicating that most cyanides are involved in the polymerization. However, there is still a small amount of cyanide group in the polymer, due to the steric hindrance caused by the polytriazine structure preventing some of the cyanide group from entering the reaction. Due to the formation of three abundant aromatic heterocyclic structures in the polymerization process, the PN resin has excellent thermal stability and thermal–mechanical properties.

The NMR spectra provided information on the molecular backbone of the monomer, which was analyzed and compared with the results of previous studies to demonstrate the successful synthesis of the monomer, whereas the FTIR spectra provided information on the characteristic groups of the monomer and the resin. In contrast to previous studies of PN resins [24,30], it was demonstrated that the synthesized resins mainly exist in three structures of triazine, phthalocyanine, and isoindoline rings, which are correspondingly analyzed in the above discussion.

**Figure 7:**
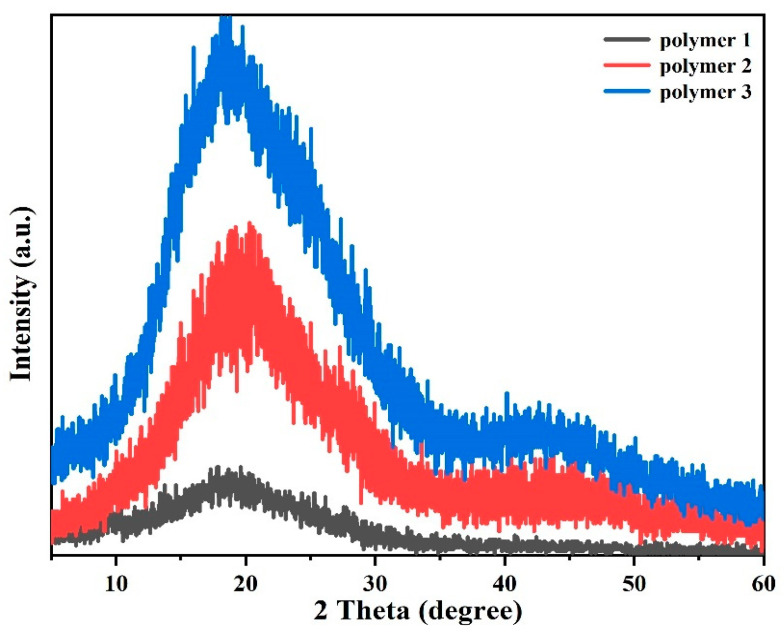
WAXD pattern of DPTP polymers.

WAXD spectra of the three polymers are shown in Figure 7, depicting relatively wide diffraction peaks, indicating that the polymers have an amorphous structure in nature. Amorphous humps of Polymer 1, Polymer 2, and Polymer 3 were observed at 2 θ = 17.88°, 20.30°, and 19.46°, respectively. The angle of the WAXD peak of amorphous diffraction represents the specific interatomic distance (R) of the material in the disordered material, which can be calculated by the formula as follows:

(1) $$R=\frac{5}{4}\;\times \;\frac{\lambda }{2\;\sin\theta }=1{.25\;d}_{\mathrm{Bragg}}$$

where λ represents the wavelength of the X-ray (Cu target, 0.15406 nm) and 2 θ on behalf of the diffraction angle, and the interatomic distance (R) is equal to 1.25 times the distance between d_Bragg_. Therefore, the interatomic distances of polymers were calculated to be 0.62 nm (Polymer 1), 0.55 nm (Polymer 2), and 0.57 nm (Polymer 3), respectively, and the atomic spacing of Polymer 2 is smaller than that of Polymer 1. The results indicate that Polymer 2 and Polymer 3 are more closely cross-linked, which may be due to the presence of nitrile groups in APPH and ANP monomers, and the nitrile group of APPH and ANP also participates in the reaction during curing.

### 3.2. Analysis of Machining Properties 

**Figure 8:**
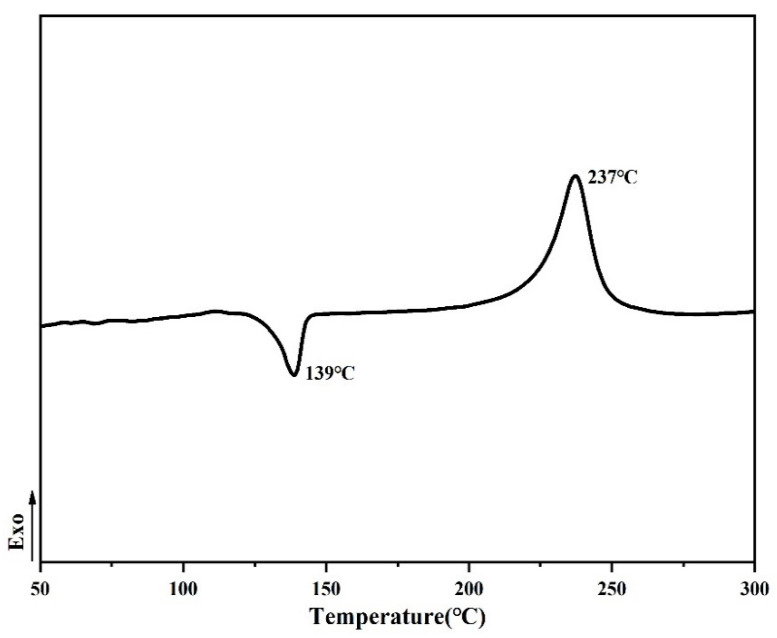
DSC of the ANP monomer.

**Figure 9:**
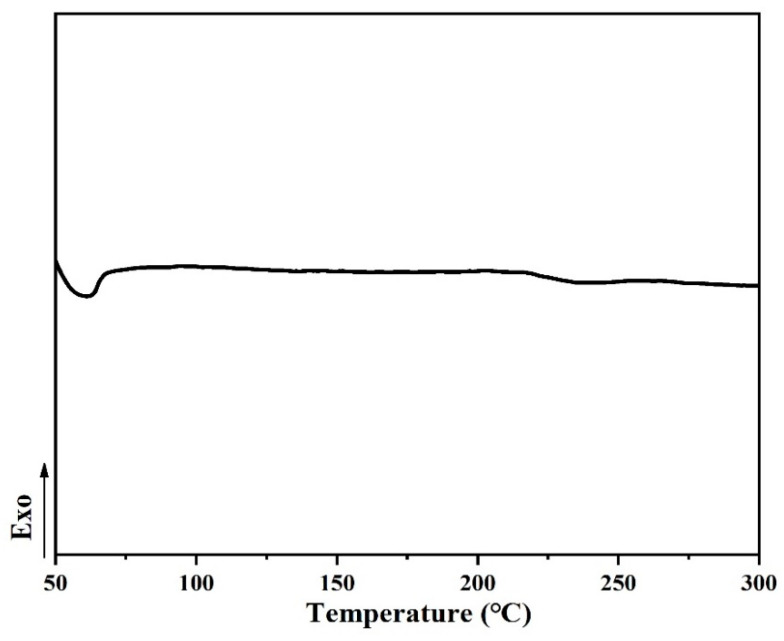
DSC of the DPTP monomer.

**Figure 10:**
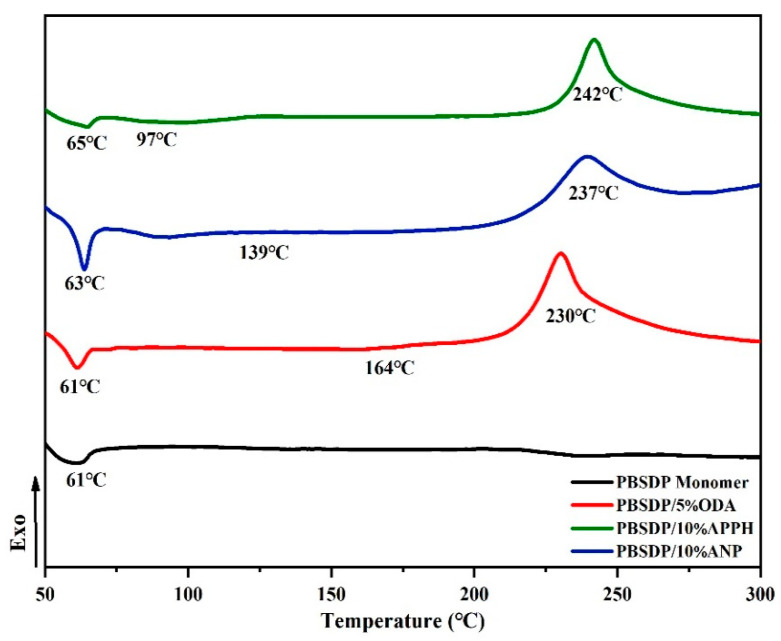
DSC of the DPTP monomer, DPTP-5%ODA mixture, DPTP-10%APPH mixture, and DPTP-10%ANP mixture.

The machining properties of the monomer including the *T*_m_ and processing window analyzed by differential scanning calorimetry (DSC) are shown in Figure 8, Figure 9 and Figure 10 when the rate of heat addition was 10 °C min^−1^. The difference between *T*_m_ and curing temperature is defined as the processing window, which has a great influence on the processing properties of PN monomer. The self-promoted cure behavior and processability of ANP shown in Figure 8 were studied by DSC. An endothermic transition that corresponds to *T*_m_ and another exothermic transition peak appears at 237 °C, which is attributed to the self-catalyzed curing reaction between cyano and amino groups, and the processing window of ANP is 98 °C. Figure 9 shows a melting endothermic peak corresponding to the *T*_m_ at 61 °C, without other phenomena of absorption and exotherm.

Figure 10 presents the DSC of the DPTP monomer, DPTP-5% ODA mixture, DPTP-10% APPH mixture, and DPTP-10% ANP mixture. Endothermic peaks appeared at 61 °C, 65 °C, and 63 °C when the DPTP monomer was cured with ODA5%, APPH10%, and ANP10% as curing agents; 164 °C, 97 °C, and 139 °C correspond to the *T*_m_ values of ODA, APPH, and ANP, respectively. The exothermic peaks of the monomer and curing agent at 230 °C, 242 °C, and 237 °C are due to the exothermic effect of curing agent on the curing reaction. Therefore, the processing window of DPTP is 169–177 °C. The results show that the *T*_m_ of DPTP monomer is greatly reduced by the introduction of sulfide, and the processing window of DPTP monomer is wider than that of numerous PN monomers previously reported [19,21,24,28,30]. Synthetic PN monomer-curing agent blends have a wider processing window than various monomer-curing agent composition systems and some self-promoted monomers. Comprehensively, the synthesized monomer demonstrated splendid processability with a low melting point and a wide processing window. 

**Figure 11:**
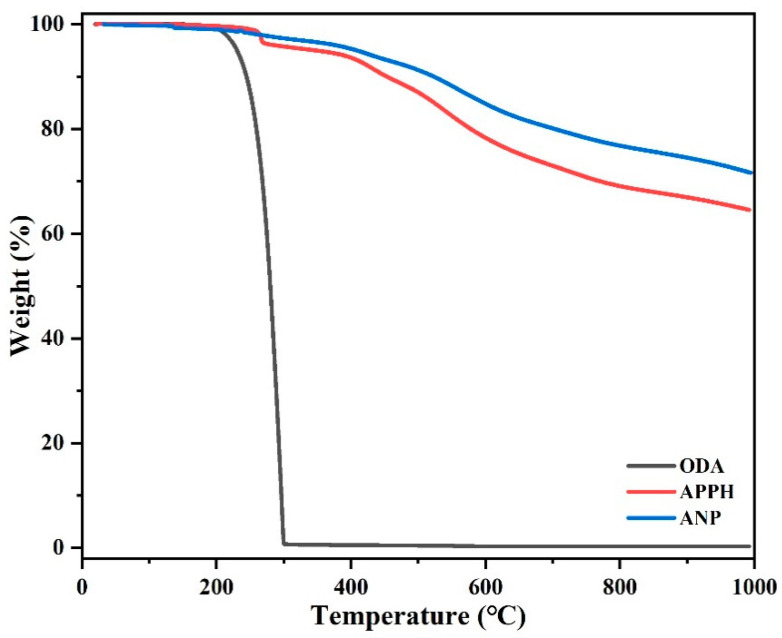
TGA of ODA, APPH, and ANP in a N_2_ atmosphere.

ANP, as an autocatalytic monomer, can not only self-polymerize, but also act as a curing agent to initiate polymerization. To determine the volatility of small-molecule curing agents, TGA tests were conducted with ODA, APPH, and ANP small-molecule curing agents as examples. As shown in Figure 11, the *T*_5%_ of the ODA small-molecule curing agent was 230 °C, and *T*_10%_ was 244 °C, which decomposed rapidly with the increase in temperature, and the carbon residue rate rapidly decreased to 0% at about 300 °C. *T*_5%_ of the APPH small-molecule curing agent was 348 °C, and *T*_10%_ was 453 °C. As the temperature rose, APPH still decomposed slowly until the carbon residue rate was close to 64%. For ANP, the *T*_5%_ of ANP monomer was 409 °C, *T*_10%_ was 523 °C, and the carbon residue rate was 73% at 1000 °C, indicating that ANP can exist stably within the curing temperature of 350 °C. Therefore, it can effectively solve the problem of volatilization of an aromatic amine curing agent at high temperature, thus reducing the influence of curing agent volatilization.

### 3.3. Study on the Kinetics of the Curing Reaction of the Monomer

The polymerization reaction in this study is bulk addition polymerization, which is a type of free radical polymerization [37]. Bulk polymerization is polymerization without any other medium, only the monomer is initiated by initiator, heat, light, radiation, etc. Gaseous, liquid, and solid monomers can be subjected to bulk polymerization.

**Figure 12:**
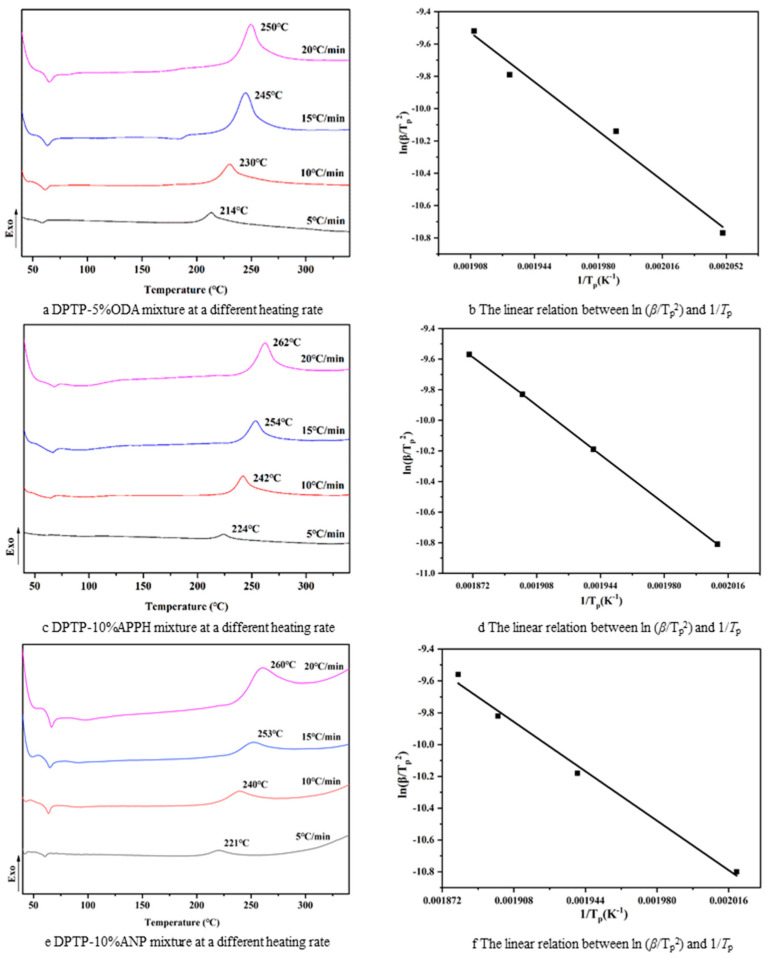
DSC curves and parameters of the monomer curing agent mixture.

Cross-linking reactions under different heating rates were assessed using scanning calorimetry with unequal temperature differences; dynamic data could be calculated through the Kissinger equation at different heating rates, and the corresponding kinetic results were obtained. As shown in Figure 12, the DSC curves of DPTP monomer reaction cured with three curing agents were derived at different heating rates of 5, 10, 15, and 20 °C min^−1^, respectively. The results show that the faster the heating rate, the higher the curing temperature. The apparent activation energy (*E*_a_) can characterize the difficulty of curing reaction calculated by the Kissinger equation:

(2) $$-\ln\frac{\beta }{T_{P}^{2}}=\frac{E_{a}}{{RT}_{P}}-\ln\frac{A^{\prime }R}{E_{a}}$$

where *β* is the heating rate (°C min^−1^), *T*_P_ is the peak temperature (K) of the exothermic curing reaction, R is the ideal gas constant (8.314 J mol^−1^ K^−1^), and *E*_a_ is the apparent activation energy. Table 2 shows the relationship between ln (*β*/*T*_P_^2^) and 1/*T*_P_ at different heating rates. Figure 12 shows the DSC curves and the linear relationship between ln (*β*/*T*_P_^2^) and 1/*T*_P_ of three different monomer and curing agent mixtures; the slope is (−*E*_a_/*R*). After calculation, the reaction activation capacity of the ODA5% curing DPTP monomer is 70.41 kJ mol^−1^, the *E*_a_ of the APPH10% cured DPTP monomer is 73.78 kJ mol^−1^, and that of the ANP10% cured DPTP monomer is 71.86 kJ mol^−1^, similar to the thermosetting resin previously reported.

### 3.4. Study on Thermomechanical Properties of Resin

**Figure 13:**
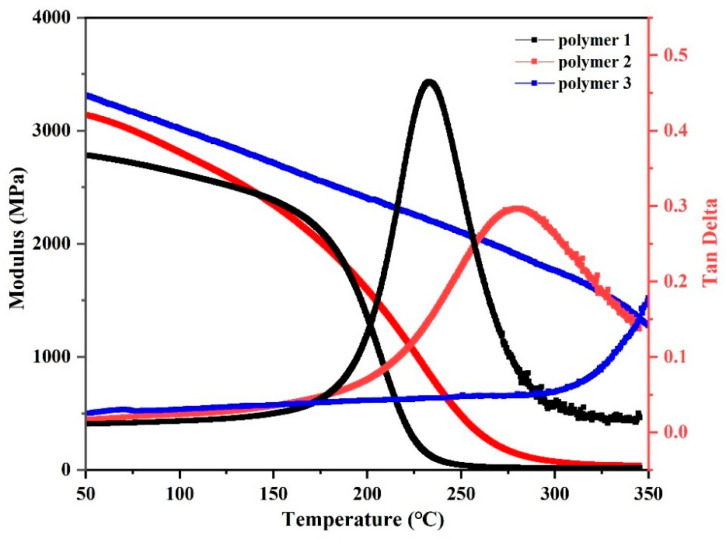
DMA of DPTP polymers.

The DPTP polymer was subjected to dynamic thermo-mechanical analysis (DMA) in single cantilever mode in a nitrogen atmosphere with a ramping rate of 5 °C min^−1^ and a temperature range of 50–350 °C. DMA was performed to prospect the thermo-mechanical properties and *T*_g_ of the polymer shown in Figure 13 and Table 3, which manifested the storage modulus (*E’*) and loss angle tangent (Tan δ). Based on the above results, the energy storage moduli of Polymer 3, 2, and 1 at 50 °C were 3315 MPa, 3140 MPa, and 2783 MPa, respectively. The energy storage modulus decreased gradually with the increase in temperature; the thermo-mechanical properties of Polymer 3 were the best, and Polymer 1 was the worst. The nitrile group in APPH and ANP structures can cross-link with the DPTP monomer; therefore, the polymer exhibited better crosslinking. The maximum tangent of the Tan δ curve represents the *T*_g_ of the polymer, which determines the upper limit of the application temperature of thermosetting resin. The corresponding *T*_g_ values of Polymer 2 and Polymer 1 were 280 °C and 234 °C, respectively, with Polymer 3 having a *T*_g_ value of more than 350 °C. The results show that for the DPTP monomer, the PN resin with better thermal–mechanical properties can be obtained with 10% ANP as the curing agent.

**Figure 14:**
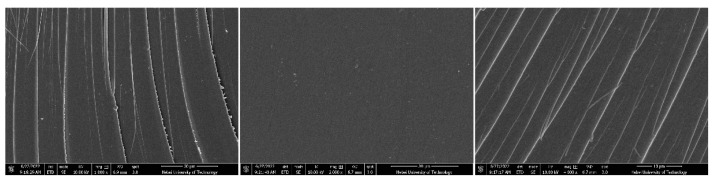
SEM of DPTP polymers.

The fracture surface scanning electron microscopy (SEM) photographs of DPTP polymer under different magnifications are shown in Figure 14. It can be seen from the figure that due to the additional polymerization mechanism of PN resin, as well as no solvent during the polymerization process, there is no gap in the whole region, which is more suitable for preparing thicker structures or components, showing obvious advantages in terms of processability. In addition, the fracture morphology of the polymer is easy to see, and the crack propagation is clearly distributed. Akin to a river, the cracks are almost parallel, exhibiting a typical brittle fracture.

### 3.5. Study on the Thermal Stability of Resin

**Figure 15:**
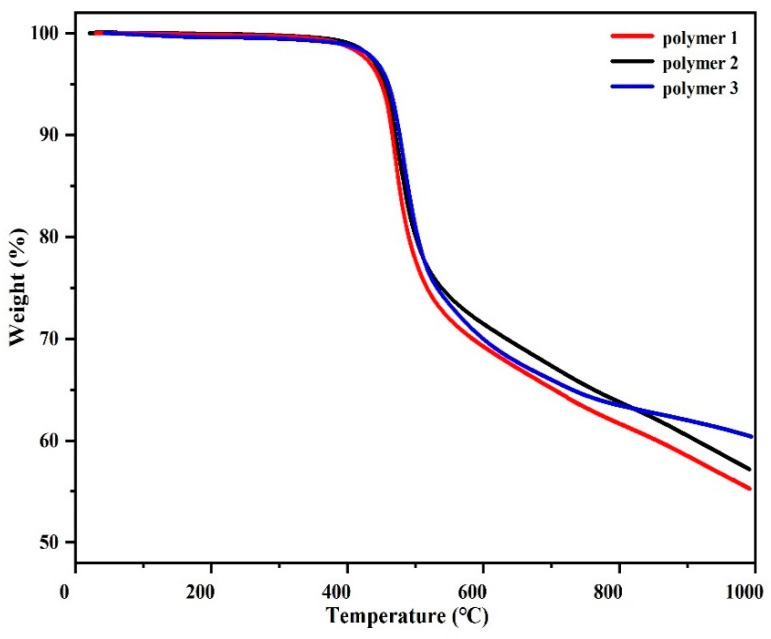
TGA of DPTP polymers in an inert atmosphere.

**Figure 16:**
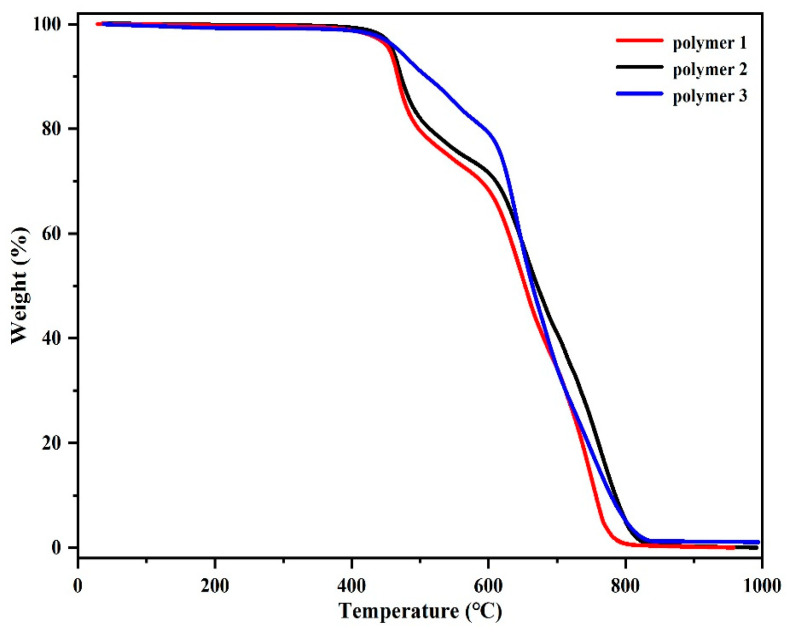
TGA of DPTP polymers in an air atmosphere.

Thermogravimetric analysis (TGA) was used to study the thermal stability of polymers in the range of 50 °C to 1000 °C in nitrogen and air atmosphere, respectively, as shown in Figure 15 and Figure 16. The characteristic data of thermal analysis, including temperature at 5% weight loss (*T*_5%_), the temperature at 10% weight loss (*T*_10%_), and carbon residue rate (CR) at 1000 °C, are summarized from the curves in Table 4. It can be seen that in a nitrogen atmosphere, the corresponding *T*_5%_ values of Polymer 3, Polymer 2, and Polymer 1 are 460 °C, 454 °C, and 450 °C, respectively, and *T*_10%_ values are 477 °C, 471 °C, and 466 °C, respectively. At 1000 °C, the carbon yields of Polymer 3, Polymer 2, and Polymer 1 were 60.4%, 57.2%, and 55.3%, respectively. In an air atmosphere, the *T*_5%_ values of Polymer 3, Polymer 2, and Polymer 1 were 469 °C, 460 °C, and 455 °C, and the *T*_10%_ values were 510 °C, 472 °C, and 467 °C, respectively. In comparison, it can be found that Polymer 3 and Polymer 2 exhibit better thermal stability than Polymer 1. The results may be attributed to the fact that the nitrile group in APPH and ANP can cross-link with the DPTP monomer. In addition, the thermal stability of Polymer 3 is stronger than that of Polymer 2; perhaps because of the introduction of naphthalene ring into the polymer and its rigid planar structure, the thermal properties of the polymer are slightly improved. 

For halogen-free polymers, according to the Van Krevelen equation, a linear correlation between the limiting oxygen index (LOI) is to measure the flame retardancy and CR′. The formula is as follows [38,39].

(3) $$\mathrm{LOI}=17.5+0.4({\mathrm{CR}}^{\prime })$$

LOI indicates the volume fraction of oxygen when the oxygen–nitrogen mixture is sufficient to sustain the combustion of sample, and CR is the coke residue rate of the polymer at 850 °C. In general, when the LOI is more than 26, the material is determined to be flame-retardant. CR’ values of Polymer 3, Polymer 2, and Polymer 1 were 62.7%, 62.2%, and 60.2%, respectively. LOI values of Polymer 3, Polymer 2, and Polymer 1 were 42.6, 42.4, and 41.6, respectively. Therefore, it can be concluded that DPTP polymer has superior flame retardancy.

### 3.6. Long-Term Stability Study

High-performance polymers have satisfactory thermal stability, little thermal decomposition reaction, and weight loss rarely at temperatures up to 300 °C. The long-term thermal stability of polymers can be assessed by thermogravimetric analysis, which investigates the thermal behavior during heating in a given environment as a function of temperature and time. In this experiment, several different polymeric materials were rapidly heated to 300 °C under nitrogen and air conditions and kept for 6 h for thermogravimetric testing to reflect the differences in thermal stability [24,40]. 

In this experiment, polypropylene (PP), epoxy resin, and poly ether ether ketone (PEEK) were used as comparison materials to compare their long-term thermal stability differences with synthetic polymers. In this case, PP was manufactured by the extrusion, granulation, and hydraulic pressure of polypropylene pellets, epoxy resin was made from epoxy resin E-51, polyether amine D230 curing agent was obtained by homogeneous mixing and hot pressing, and PEEK was purchased from Victrex UK.

**Figure 17:**
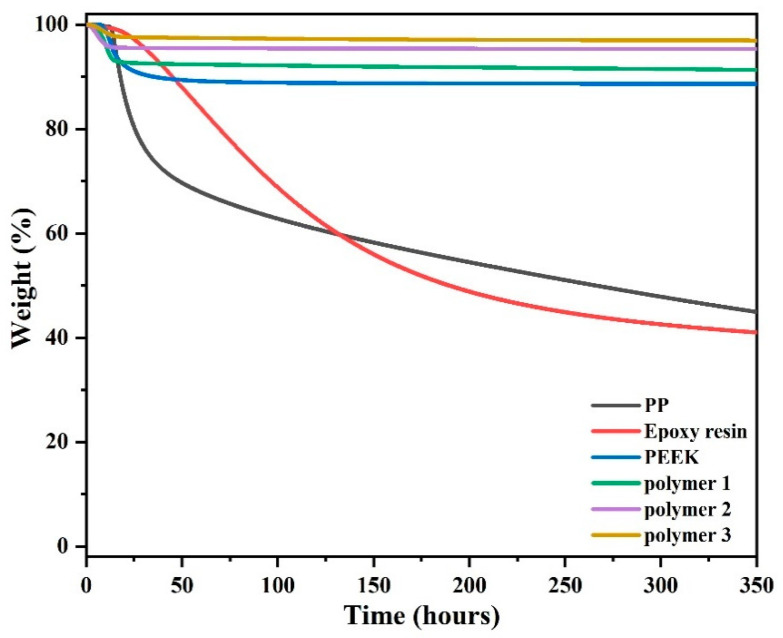
Long-term stability of five materials in an inert atmosphere at 300 °C.

**Figure 18:**
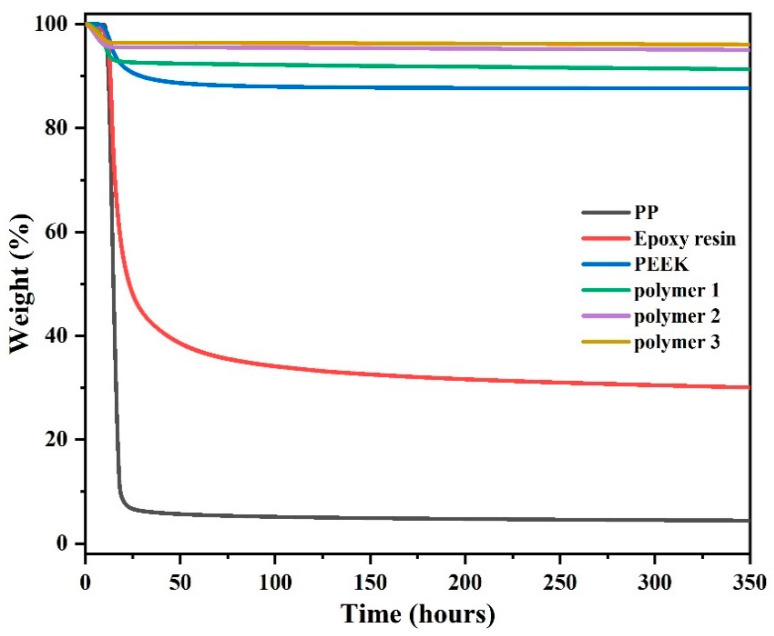
Long-term stability of five materials in an air atmosphere at 300 °C.

Figure 17 and Figure 18, and Table 5 show the thermogravimetric curves of the five polymeric materials, which reflect the difference in thermal stability of the five polymer materials when they were heated rapidly from room temperature to 300 °C and prolonged use at 300 °C for 6 h. Thus, it can be seen that Polymer 3, Polymer 2, and Polymer 1 maintained 97.0 wt.%, 95.3 wt.%, and 91.6 wt.% in a nitrogen atmosphere, respectively. As a general high-performance polymer, PEEK has strong thermal stability, maintaining 88.6 wt.% under the same conditions; however, its long-term stability is still inferior to that of the synthetic PN resin. PP and epoxy resins have much worse thermal stability than the PN polymers, with only 43.6 wt.% and 40.4 wt.% surplus, respectively, under the same conditions. In air, after 6 h at 300 °C, Polymer 3, Polymer 2, and Polymer 1 remained as 96.1 wt.%, 95.0 wt.%, and 91.5 wt.%, respectively. The thermal stability of PEEK was worse than that of PN polymers at 87.6 wt.%. Under these conditions, epoxy retained only 28.9 wt.% and PP was the least thermally stable with a residue of only 4.4 wt.% and a weight loss of 95.6 wt.%.

The long-term stability of synthetic polymers is higher than that of commonly used polymers, such as PEEK, a widely used high-performance, high-temperature-resistant, and highly thermally stable polymer. First, a result of the introduction of a pyridine ring consisting polymer of a rigid aromatic structure compensates for the loss of rigidity caused by long chains, which exhibit higher thermal stability, strength, and modulus, and have a wide processing window to meet both requirements for polymer utility. Second, polymers with cross-linked structures are more thermally stable than the corresponding linear polymers because multiple bonds must break when thermal degradation occurs. Clearly, the high-temperature stability of the polymers comes from the aromatic molecular structure and the cross-linked polymer network. This is especially true for Polymers 2 and 3, which contain cyano-curing agents to increase the cross-linked density of the polymer. In summary, the synthesized polymers with high cross-linkage have excellent thermal stability and can be used at high temperatures for long periods of time. They are thermally more stable than many widely used polymeric materials on the market, and also demonstrate the utility of synthetic polymers. 

### 3.7. Water Absorption Study 

**Figure 19:**
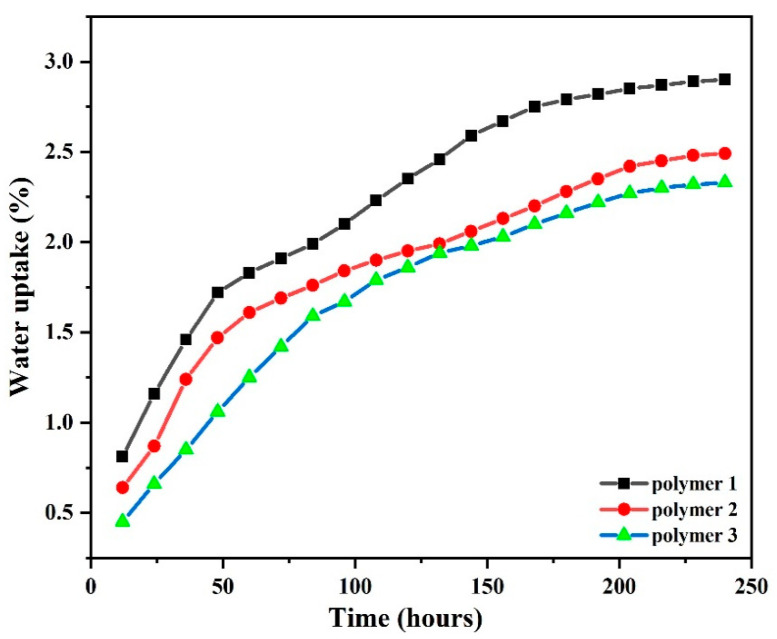
Water uptake of DPTP polymers.

By analyzing the structure of the PN resin, it can be assumed that it has outstanding water resistance and can be used in humid environments with strong performance. In this regard, the following experiments were designed to prove the idea: the polymers were polished first; then, they were immersed in a container with a sufficient amount of deionized water. They were taken out at the same time and dried with absorbent paper in order to avoid the influence of water molecules carried on the surface on the experimental results, and finally, the dried samples were weighed and recorded. The water absorption rate was calculated according to Equation (4).

(4) $$\mathrm{Water}\;\mathrm{absorption}\left(\%\right)=\frac{\left(M_{2}-M_{1}\right)}{M_{1}}\times 100\%$$

where M_1_ and M_2_ are the weights of PN resin before soaking and after absorbing water for a period of time, respectively. The water absorption curves of different sample strips with time can be calculated by Equation (4), as shown in Figure 19. The data in the figure show that the growth rate of water absorption of the PN resin in the first 100 h into the water is fast, and with the increase in soaking time the growth rate gradually becomes smaller, and the water absorption rate no longer increases after 200 h, indicating that the deionized water absorbed by the PN resin has reached saturation. The water absorption rates of PN resin strips near saturation were 2.84 wt.% (Polymer 1), 2.42 wt.% (Polymer 2), and 2.33 wt.% (Polymer 3). It can be concluded that: first, the cross-linked mesh structure of the PN resin makes it difficult for deionized water to penetrate into the sample, so the water absorption rate is significantly lower than that of other thermosetting resins; second, due to the imperfect cross-linking of the mesh structure of the PN resin, there are gaps between the molecular chains due to the presence of site resistance in the space, so the sample strips still have a small amount of water absorption; third, the water absorption rate of the PN resin decreases with the post-curing time and temperature. This is in accordance with the curing kinetic equation.

## 4. Conclusions

The PN monomer (DPTP) with sulfide bond and pyridine structure was successfully promoted by nucleophilic substitution reaction using 4,4′-dihydroxy diphenyl sulfide, 2,6-dichloropyridine, and 4-nitrophthalonitrile as raw materials. The structure of DPTP was characterized by NMR and FT-IR. DSC analysis demonstrated that the *T*_m_ of DPTP was 61 °C, the processing window was about 170 °C, and the processing range was very wide. The curing behavior of DPTP monomers with different curing agents was studied by DSC scanning at diverse heating rates, with the apparent activation energies calculated by Kissinger’s method. TGA showed that *T*_5%_ of the DPTP monomer was 460 °C in nitrogen. In addition, DMA manifested that the storage modulus of DPTP at 50 °C was 3315 MPa. Therefore, DPTP has admirable thermal stability and satisfactory thermo-mechanical properties, which stands out in polymer materials, and is a potential candidate for advanced composite matrix, adhesive, and microelectronic applications [41,42,43].

## Figures and Tables

**Table 1:** Curing process of PN resin.

Polymer	Curing Process	Curing Agent
**Polymer 1**	200 °C × 2 h+230 °C × 2 h + 260 °C × 4 h + 290 °C × 4 h + 320 °C × 4 h + 350 °C × 4 h	ODA5%
**Polymer 2**	200 °C × 2 h+230 °C × 2 h + 260 °C × 4 h + 290 °C × 4 h + 320 °C × 4 h + 350 °C × 4 h	APPH10%
**Polymer 3**	200 °C × 2 h + 230 °C × 2 h + 260 °C × 4 h + 290 °C × 4 h + 320 °C × 4 h + 350 °C × 4 h	ANP10%

**Table 2:** The values of ln(*β*/*T*_p_^2^) and 1/*T*_p_.

*β* (°C min^−1^)	*T*_p_(K)_1_	ln(*β*/*T*_p_^2^)_1_	1/*T*_p_(K^−1^)_1_	*T*_p_(K)_2_	ln(*β*/*T*_p_^2^)_2_	1/*T*_p_(K^−1^)_2_	*T*_p_(K)_3_	ln(*β*/*T*_p_^2^)_3_	1/*T*_p_(K^−1^)_3_
5	487.15	−10.77	2.05 × 10^−3^	497.15	−10.81	2.01 × 10^−3^	494.15	−10.80	2.02 × 10^−3^
10	503.15	−10.14	1.99 × 10^−3^	515.15	−10.19	1.94 × 10^−3^	514.15	−10.18	1.94 × 10^−3^
15	518.15	−9.79	1.93 × 10^−3^	527.15	−9.83	1.90 × 10^−3^	525.15	−9.82	1.90 × 10^−3^
20	523.15	−9.52	1.91 × 10^−3^	535.15	−9.57	1.87 × 10^−3^	533.15	−9.56	1.88 × 10^−3^

**Table 3:** Thermal–mechanical properties of the DPTP PN resin.

Sample	Storage Modulus (MPa) *T* = 50 °C	*T_g_* (°C)
Polymer 1	2783	234
Polymer 2	3140	280
Polymer 3	3315	>350

**Table 4:** Thermal parameters of DPTP polymers in inert and air atmospheres.

	In Nitrogen			In Air		
	*T*_5%_/°C	*T*_10%_/°C	CR/%	*T*_5%_/°C	*T*_10%_/°C	CR/%
Polymer 1	450	466	55.3	455	467	-
Polymer 2	454	471	57.2	460	472	-
Polymer 3	460	477	60.4	469	510	-

**Table 5:** Long-term stability data for several polymers in inert and air atmospheres.

	PP	EP	PEEK	Polymer 1	Polymer 2	Polymer 3
In nitrogen	43.6%	40.4%	88.6%	91.6%	95.3%	97.0%
In air	4.4%	28.9%	87.6%	91.5%	95.0%	96.1%

## Data Availability

The data presented in this study are available on request from the corresponding author.
